# The DMT1 IVS4+44C>A polymorphism and the risk of iron deficiency anemia in children with celiac disease

**DOI:** 10.1371/journal.pone.0185822

**Published:** 2017-10-12

**Authors:** Carlo Tolone, Giulia Bellini, Francesca Punzo, Alfonso Papparella, Erasmo Miele, Alessandra Vitale, Bruno Nobili, Caterina Strisciuglio, Francesca Rossi

**Affiliations:** 1 Department of Woman, Child and of General and Specialist Surgery, University of Campania “Luigi Vanvitelli”, Naples, Italy; 2 Department of Experimental Medicine, University of Campania “Luigi Vanvitelli”, Naples, Italy; 3 Department of Translational Medical Science, Section of Pediatrics, University of Naples “Federico II”, Naples, Italy; Hospital Israelita Albert Einstein, BRAZIL

## Abstract

**Background:**

Iron deficiency anemia in celiac disease is related to impaired duodenal mucosal uptake, due to villous atrophy. Iron enters the enterocytes through an apical divalent metal transporter, DMT1. Different DMT1 transcripts have been identified, depending on the presence of an iron-responsive element that allows DMT1 up-regulation during iron starvation. An intronic DMT1 polymorphism, IVS4+44C>A, has been associated with metal toxicity, and the CC-carriers show high iron levels.

**Aims:**

This study investigates the association between DMT1 IVS4+44C>A and anemia in a cohort of 387 Italian celiac children, and the functional role of the polymorphism.

**Methods and results:**

By association analysis, we found that DMT1 IVS4+44-AA genotype confers a four-fold risk of developing anemia, despite of atrophy degree. By analysis of mRNA from gastroesophageal biopsies, we found that total DMT1 is significantly upregulated in presence of mild, but not severe, atrophy, independently from IVS4+44C>A variant, and in normal but not in atrophic CC-biopsies. Moreover, we found that A-allele is associated to preferential expression of the DMT1 transcripts lacking the iron-responsive element, thus limiting the DMT1 overexpression that normally occurs to respond to iron starvation.

**Discussion:**

Possibly, the IVS4+44-AA-related dysregulation of the iron-induced changes in DMT1 expression is not able to impair iron absorption in physiological condition. However, if exacerbated by the concomitant massive loss of functional absorbing tissue paralleling worsened stages of villus atrophy, it might be ineffective in counteracting iron deficiency, despite of DMT1 overexpression.

**Conclusion:**

We suggest, for the first time, that celiac disease may unmask the contribution of the DMT1 IVS4+44C>A polymorphism to the risk of anemia.

## Introduction

Celiac disease (CD) is a chronic inflammatory disease of the small bowel that occurs with the ingestion of gluten. Although HLA-DQ2 variant is required for the gluten-derived peptide gliadin presentation to T-cells, non-HLA genetic factors account for the majority of heritable risk [1] and for the predisposition of CD patients to develop other disorders [2, 3].

Our study was born to investigate the contribution of non-HLA genetic factors in the development of iron deficiency anemia (IDA) in children with CD.

Iron is an important micronutrient that may be depleted in celiac disease. Indeed, IDA is often recorded in newly diagnosed CD [3, 4] and may persist for variable periods after initiation of a gluten-free diet [5]. IDA may complicate well-established CD, but may also be the presenting clinical feature in the absence of classical symptoms such as diarrhea or weight loss [6–8].

Unfortunately, this interesting relationship between IDA and CD has been poorly studied [9].

Although detailed epidemiological data on IDA in CD are limited, recent studies suggest that IDA may be significant in both CD children [10, 11] and adults [12].

Historically IDA observed in CD has been ascribed to impaired duodenal mucosal uptake due to reduced absorptive surface in the proximal small intestine area, as result of villous atrophy. This reflects, in large part, the prominent duodenal mucosal geographic distribution of CD and the concurrence with the principal site of iron uptake from enterocytes of the small intestine. However, only 10% of dietary iron is absorbed to meet body iron requirement [13]. Therefore, considering this redundancy in absorptive capacity, it would seem that reducing the available surface area might not be sufficient to explain the IDA observed in CD. Moreover, a significant proportion of CD patients remain iron depleted despite the disease control and their clinical responsiveness to a gluten free diet [4]. A potential reason for this disparity could be justified from an alteration of the iron metabolism regulatory proteins.

Iron enters the epithelial cell of the duodenal mucosa in ferrous form through the apical or brush border divalent metal transporter DMT1 [14]. The efficiency of iron absorption parallels the level of DMT1 expression [15]. Rodents carrying a missense mutation in DMT1 (Gly-185-Arg), suffer from a recessively inherited systemic iron deficiency and from microcytic anemia [16, 17]. A greater expression of DMT1 is found in iron deficient rats and less in iron-loaded animals than in controls. Moreover, in presence of iron deficiency, DMT1 increases the spanning of the entire brush border membrane instead of limiting its localization to villus apical region [18]. Indeed, different DMT1 transcripts have been identified, depending on the presence of an iron responsive element (IRE) at the 3’-UTR. DMT1 IRE containing transcript (DMT1 +IRE) allows to modulate DMT1 expression according to iron load, whereas DMT1 -IRE does not respond to iron levels fluctuation [19].

Microcytic anemia caused by DMT1 mutations has been also identified in human subjects [20].

In addition, an intronic DMT1 polymorphism, DMT1 IVS4+44C>A, has been associated with increased risk for Wilson’s disease [21], age related macular degeneration [22], Parkinson's disease [23], and, more recently, also with inter-individual variations in blood iron levels [24]. In all of these studies, the C-allele was more frequent in patients in comparison to the control group and seemed to be a risk factor for these diseases, possibly increasing the susceptibility to metal toxicity [25]. Indeed, homozygous C-allele carriers showed statistically higher iron and lead levels than AA and CA subjects [24]. A part from this latter study, the effect of DMT1 IVS4+44C>A polymorphism on iron metabolism needs to be elucidated.

Interestingly, the DMT1 iron transporter is known to be upregulated in celiac disease to counteract villous atrophy [26].

Taking advantage from these evidences, we tried to explore the possibility that the variants of the polymorphism could differently modulate the DMT1 changes in response to the villous atrophy.

Therefore, the aim of our study was to investigate the association between the DMT1 IVS4+44C>A polymorphisms and IDA in our cohort of children with CD and to possibly clarify the functional role of this intronic variant.

## Material and methods

### Cohort description

The molecular study was carried out on 387 unrelated celiac children [age 5.07±4.00; females 235 (61%)] from Southern Italy referred from March 2008 to December 2015 to the Department of Woman, Child, and General and Specialist Surgery of the University of Campania “Luigi Vanvitelli”. Demographic and clinical data are reported in S1 Table. A cohort of 164 healthy children from the same geographic area was used as control group for Hardy-Weinberg equilibrium (HWE) calculation, since there aren’t existing meta-analysis data for DMT1 IVS+44C>A expected frequencies or DMT1 IVS4+44C>A frequency data from Southern Italy.

For CD diagnosis, we performed upper-gastrointestinal endoscopy and duodenal biopsies in all the recruited patients [27]. Patients were divided in anemic and not anemic considering hemoglobin (Hb) values lower or higher than 3° percentile [28] (Table 1).

**Table 1:** Clinical features of 387 Italian children with Celiac Disease stratified according to IDA.

	IDA	non-IDA	*p*-value ^a^^,^^b^
**Patients, n (%)**	134 (35)	253 (65)	
**Males, n (%)**	67 (50)	85 (34)	**0.0021** ^a^
**Females n (%)**	67 (50)	168 (66)	
**Age, years, median (range)**	2.5 (0.58–15.00)	4.33 (0.75–18)	**0.00009** ^b^
**z-score BMI, median (range)**	-0.36 (-2.41–7.73)	0.083(-4.42–2.34)	**0.004** ^b^
**Hb, g/dl, median (range)**	10.7 (5.6–12.1)	12.4 (11–15.3)	**<10**^**−10**^ ^b^
**MCV, fl, median (range)**	70.6 (50.2–92.5)	79.3 (38.5–91.1)	**<10**^**−10**^ ^b^
**Sideremia, mcg/dl, median (range)**	30 (6–273)	63 (10–168)	**<10**^**−10**^ ^b^
**Ferritin, ng/dl, median (range)**	11 (1–134)	20 (3–124)	**2.7**x**10**^**-7**^ ^b^
**Transferrin, mg/dl, median (range)**	331 (198–669)	292.5 (166–999)	**2.9**x**10**^**-6**^ ^b^
**Saturation Index, median (range)**	0.07 (0.017–0.698)	0.17 (0.018–0.447)	**<10**^**−10**^ ^b^
**SI < 0.10**	86 (64)	53 (21)	**<10**^**−4**^ ^a^
**SI ≥ 0.10**	48 (36)	200 (79)	
**Anti-tTg, U/ml, median (range)**	24.8 (0.01–202)	30.7 (0.03–824)	0.46 ^b^
**AGA IgA, U/ml, median (range)**	18.5 (0.01–372)	9.29 (0.22–415)	0.18 ^b^
**AGA IgG, U/ml, median (range)**	52.3 (0.33–856)	22.83 (0.42–821)	0.06 ^b^
**EMA, U/ml, n (%)**			0.72 ^a^
present	132 (98)	247 (98)	
absent	2 (2)	6 (2)	
**Villous Atrophy, n (%)**			0.61 ^c^
3a	23 (17.5)	40 (16)	
3b	41 (30.5)	90 (35)	
3c	70 (52)	123 (49)	

^a^ Fisher’s exact test and

^c^ chi-square test to determine the independence between the frequency of occurrence of the categorical variables and IDA presence

^b^ Mann-Whitney test for medians comparison of not normally distributed continuous variables of IDA and non-IDA samples; the range is reported as minimum and maximum values. *p*<0.05 (or *p*<0.0045 after Bonferroni correction) has been considered significant.

Data have been normalized only for z-score BMI, since Hb<3° percentile (IDA presence) is already related to age and sex.

Abbreviations: BMI, body mass index; Hb, hemoglobin; MCV, mean corpuscular volume; SI, saturation index; Anti-tTg, tissue transglutaminase antibodies; AGA, anti-gliadin antibody; Ig, immunoglobulin; EMA, anti-endomysial antibody.

### Ethical considerations

The Ethic Committee of the University of Campania “Luigi Vanvitelli” approved the study (protocol registration number #1312). Written informed consent from parents and assent from children were obtained. All the procedures were in accordance with the World Medical Association’s Declaration of Helsinki (1964 and its later amendments).

The participant-level data (individual data points behind means, medians and variance measures presented in the results and some of the Tables) were not included in the supporting information due to the patient privacy, according to the written informed consent and to the approved study protocol. Data are available on specific request applied to the Ethic Committee of the University of Campania “Luigi Vanvitelli” (Comitato Etico AOU “Luigi Vanvitelli”; Piazza Luigi Miraglia, 2–80138 Napoli; +39800177780; protocollo@policliniconapoli.it).

### Molecular screening

Genomic DNA was extracted from whole blood (ISOLATE II, Genomic kit, Bioline, USA). To identify DMT1 IVS4+44C>A polymorphism, DNA was amplified with specific primers (Primer3; http://bioinfo.ut.ee/primer3-0.4.0/primer3/), and amplimers were directly sequenced (ABI PRISM 3100, Applied Biosystem, Foster City, CA, USA).

### RNA isolation from duodenal biopsies and RT-PCR

Duodenal biopsies were taken from 27 consecutive subjects who underwent upper-gastrointestinal endoscopy for suspected CD (n = 22) or gastroesophageal reflux disease (n = 5). Patients were divided into 4 groups according to villous atrophy degree [25]: T0, T3a, T3b, T3c.

Total RNA was isolated using Trizol (Quiagen, Germany) after tissue homogenization (ULTRA-TURRAX T8, IKA-WERKE, Germany). RNA purity and integrity were assessed by UV spectrophotometer (Nano-Drop ND 1000, NanoDrop Technologies, LLC, Wilmington, USA). The cDNA was retro-transcripted from 250 ng total RNA (One-Step RT-PCR kit, Bioline, USA).

#### Real-time PCR for total DMT1 quantification

Two serial 10x cDNA dilutions were used to quantify total DMT1 levels respect to the housekeeping β-actin. Assay was performed in triplicate. Twenty-five μl reaction contained: 2 μl cDNA, 12.5 μl SYBR-green Master Mix (Bio-Rad, Hercules, CA, USA) 10 μl primers mix [20 μM] (Primer3-0.4.0). The thermal cycling program was: 95°C-10’, followed by 40 cycles of 95°C-15” and 60°C-1’. Data were analyzed by Icycler software (Bio-Rad, Berkeley, USA), using the comparative cycle threshold (Ct) method (2^-ΔΔCt^).

#### Semi-quantitative PCR for eof DMT1 +IRE/-IRE transcripts ratio

Fifty nanograms of cDNA were selectively amplified for DMT1 +IRE and–IRE transcripts, as previously described [19], and for the ribosomal protein S18 (RPS18), as housekeeping gene. Amplimers, resolved into 2.0% agarose gel, were detected by “Gel Doc 2000 UV System” (Bio-Rad, Hercules, CA, USA). The quantification of the pixels of all the bands of the amplimers was made by using the quantization software of the same instrument (Quantity One—4.6.5, Bio-Rad, Hercules, CA, USA). The quantification of the +IRE and–IRE bands was performed with respect to the S18 bands, by dividing the signal of the IRE bands for the signal of their relative S18 bands. After normalization with respect to S18, the ratio between +IRE/18S and–IRE/18S values was calculated. These final data were categorized as less or more than one.

### Statistical analysis

All the analyses were conducted by using Statgraphics CENTURION XV.II (Adalta, Arezzo, Italy; STATPOINT TECHNOLOGIES INC., Virginia, USA).

Clinical data were checked for normal distribution and reported as median with range values if not normally distributed continuous variables, or were categorized if discrete.

To evaluate differences between clinical continuous variables it was used the nonparametric test of Mann-Whitney if considering two different groups or the nonparametric Kruskal-Wallis test when considering three different groups.

The Fisher’s exact test or the chi-square test was used to evaluate the difference among categorical variables distribution. The Fisher’s exact test was used in a 2x2 contingency table. The chi-square test was used in a 2x3 or 3x2 contingency table only if the expected frequency was 5 or greater for at least 80% of the cells, and there was not expected frequency smaller than 1.0, otherwise a Yates’ correction for the *p*-value was considered.

Data were normalized for age, sex, and z-score BMI. Data in Table 1 were normalized only for z-score BMI, since the presence of IDA is considered when Hb is less than the third percentile for age and sex. A *p*-value less than 0.05 was considered significant. Nevertheless, where we tested many different variables, we applied a Bonferroni correction for the significance cut-off level.

Molecular data were represented as mean ± SD of the fold change [2^-ΔΔCt^ where ΔΔCt = ΔCt _Target Sample_—ΔCt _Reference Sample_]. Nevertheless, due to the small number size, to evaluate differences between groups we analysed the ΔCt values (Ct Target Gene−Ct _Reference Gene_) using the unpaired t-test for two groups and the one-way ANOVA for more than two groups.

#### Logistic regression

To confirm if the DMT1 IVS4+44C>A represented the best fitting biologically reasonable model that determine IDA presence (or not) in celiac patients, we used a logistic regression, considering as explanatory variables the degree of villous atrophy for the celiac disease-related effects on iron uptake and the z-score BMI for the obesity-related effects on iron metabolism. Beyond DMT1 IVS4+44C>A variant, all the other significant variables at the univariate analysis (Table 1) could not be considered as predictors since they are by definition clinical and biological signs of anemia.

#### Odd ratio

To possibly demonstrate that DMT1 IVS+44C>A variant can modulate the DMT1 +IRE/–IRE transcript ratio, we combined the AA and CA biopsy groups together due to the small sample size of the AA group (n = 2), and compared this AA+CA biopsy group toward the CC group. Therefore, given the A-allele presence, we calculated the odds that the DMT1 +IRE/–IRE transcript ratio was less than 1, compared to the odds that DMT1 –IRE was more expressed with respect to DMT1 +IRE in CC-carriers.

## Results

### Association between DMT1-IVS4+44C>A polymorphism and anemia in CD patients

We found significant differences in Hb levels, mean corpuscular volume (MCV) values, and all the parameters related to iron absorption between the IDA and the non-IDA celiac patients. There was no difference in mean level of specific antibodies or in severity of villus atrophy between the two groups. The anemic patients were significantly younger and were more frequently males (Table 1).

Genotyping patients for the DMT1 IVS4+44C>A polymorphism resulted in 161 CC-homozygous, 193 CA-heterozygous and 33 AA-homozygous patients. The allelic frequencies were 0.67 for the C-allele and 0.33 for the A-allele. Genotyping controls for DMT1 IVS4+44C>A resulted in 66 CC-homozygous, 81 CA-heterozygous and 17 AA-homozygous patients. The control allelic frequencies were 0.65 for the C-allele and 0.35 for the A-allele. No significant difference was observed between the case and control groups both for the allelic frequencies (χ^2^ = 0.263, df = 1, *p* = 0.61) and the genotype distribution (χ^2^ = 0.486, df = 2, *p* = 0.78). There was no significant difference between observed and expected genotypes of the patients (161-CC, 193-CA, 33-AA vs. 173-CC, 171-CA, 42-AA; χ^2^ = 2.84, df = 2, *p* = 0.24). Moreover, no difference was found between observed genotypes and expected genotypes in patients also by using allele frequencies from the control group (161-CC, 193-CA, 33-AA vs. 164-CC, 176-CA, 47-AA; χ^2^ = 3.26, df = 2, *p* = 0.2). The polymorphism was in Hardy-Weinberg equilibrium.

Stratifying the clinical features according to the DMT1 IVS4+44C>A polymorphism, we observed an association between the AA genotype and the Hb values lower than 3° percentiles (*p* = 0.0002; Table 2). A-allele carriers and homozygous AA patients showed an increased risk for presenting anemia of about two folds and four folds, respectively (*p* = 0.006 Table A in S1 File and *p* = 0.0002; Table B in S1 File). The AA genotype was also associated with significant lower MCV and sideremia, and with significant lower transferrin saturation index (SI) respect to CC and CA genotypes (Table 2). In particular, AA homozygous patients showed an increased risk for SI<10% of more than three folds (*p* = 0.008). Although not significant, decreased levels of ferritin and increased levels of transferrin were also found in AA respect to those found in CA and CC patients. No association was observed with specific antibodies levels or with the degree of villous atrophy (Table 2).

**Table 2:** Clinical features of 387 Italian children with Celiac Disease stratified according to DMT1 IVS+44C>A polymorphism.

DMT1 IVS+44C>A	CC	CA	AA	*p*-value ^a^^,^^b^
**Patients, n (%)**	161 (42)	193 (50)	33 (8)	
**Males, n (%)**	63 (39)	77 (40)	12 (36)	0.93 ^a^
**Females, n (%)**	98 (61)	116 (60)	21 (64)	
**Age, years, median (range)**	3.8 (0.67–17.50)	3.9 (0.58–18.00)	2.8 (1.08–15.83)	0.10 ^b^
**z-score BMI, median (range)**	-0.3 (-3.35–2.34)	-0.13 (-4.42–2.3)	0.1 (-0.9–7.73)	0.11 ^b^
**Hb, g/dl, median (range)**	12.1 (5.9–14.8)	11.9 (5.6–15.3)	11.1 (5.9–13)	**0.00001** ^b^
**Hb < 3°, n (%)**	43 (27)	70 (36)	21 (64)	**0.0002** ^a^
**Hb ≥ 3°, n (%)**	118 (73)	123 (64)	12 (36)	
**MCV, fl, median (range)**	77.7 (53.3–87.3)	77.15 (50.2–92.5)	73.3 (38.5–91.1)	**0.017** ^b^
**Sideremia, mcg/dl, median (range)**	50.5 (55.1–62.7)	56 (55.5–62.5)	32 (34.5–51.3)	**0.03** ^b^
**Ferritin, ng/ml, median (range)**	17 (14–21.1)	17 (14–21.2)	19 (6.3–21.2)	0.40 ^b^
**Transferrin, mg/dl, median (range)**	309 (268–348)	298 (266–336)	334 (278–409)	0.18 ^b^
**Saturation Index, median (range)**	0.14 (0.09–0.17)	0.15 (0.13–0.17)	0.07 (0.05–0.12)	**0.01** ^b^
**SI < 0.10, n (%)**	65 (40)	60 (31)	21 (64)	**0.001** ^a^
**SI ≥ 0.10, n (%)**	96 (60)	133 (69)	11 (36)	
**Anti-tTg, U/ml, median (range)**	26.2 (22–50)	32.5 (25.1–66.5)	24.8 (15.6–94.1)	0.36 ^b^
**AGA IgA, U/ml, median (range)**	12.1 (4.9–18)	11.2 (6.5–23.9)	8.7 (2.8–47.1)	0.85 ^b^
**AGA IgG, U/ml, median (range)**	28.7 (14.7–59.2)	32.5 (18.3–61.3)	20.1 (7.1–64.2)	0.78 ^b^
**EMA, n (%)**				0.94 ^c^
present	158 (98)	188 (97)	32 (97)	
absent	3 (2)	5 (3)	1 (3)	
**Villous Atrophy, n (%)**				0.42 ^a^
3a	29 (18)	41 (21)	3 (10)	
3b	61 (38)	66 (34)	11 (33)	
3c	71 (44)	86 (45)	19 (57)	

^a^ chi-square or

^c^ Yates’ chi square test to determine independence between the frequency of occurrence of the categorical variables and DMT1 IVS+44C>A variants

^b^ Kruskal-Wallis test for medians comparison of not normally distributed continuous variables of the three groups; the range is reported as minimum and maximum values. *p*<0.05 *p*<0.05 (or *p*<0.005 after Bonferroni correction) has been considered significant.

Data have been normalized for age, sex, and z-score BMI.

Abbreviations: BMI, body mass index; Hb, hemoglobin; MCV, mean corpuscular volume; SI, saturation index; Anti-tTg,tissue transglutaminase antibodies; AGA, anti-gliadin antibody; Ig, immunoglobulin; EMA, anti-endomysial antibody.

A logistic regression analysis was performed to analyze the contribution of the polymorphism to IDA feature in the presence of villous atrophy. The analysis showed that DMT1 IVS4+44 AA-variant is the best predictor for the increased risk of IDA with respect to atrophy severity or z-score BMI (Table 3).

**Table 3:** Logistic regression analysis for 387 celiac patients presenting (134) or not (253) IDA (Hb less than 3° percentile).

	Estimated Regression Model			Likelihood Ratio Tests		
*Parameter*	*Estimate*	*Standard Error*	*Estimated Odds Ratio*	*chi-square*	*df*	*p-value*
CONSTANT	-1.150	0.243				
z-score BMI	-0.093	0.347	0.911	0.412	1	0.521
Villous Atrophy = 3a	0.107	0.330	1.113	0.197	2	0.906
(n = 63)						
Villous Atrophy = 3b	0.109	0.272	1.115			
(n = 131)						
DMT1 IVS+44C>A = AA	1.799	0.478	6.043	16.525	2	0.0003
(n = 33)						
DMT1 IVS+44C>A = CA	0.579	0.257	1.784			
(n = 193)						

Abbreviations: IDA, Iron deficiency anemia; Hb, Hemoglobin; df, degrees of freedom; BMI, body mass index.

Since Hb percentiles are calculated with respect to mean values referred to specific age for both genders, sex and age have not been considered as co-factors.

*p*<0.05 has been considered significant.

### DMT1 expression in duodenal biopsies

The histologic evaluation of the duodenal biopsies showed a normal gut with no atrophy in six of the 22 potential celiac subjects, as well as in the 5 patients with suspected gastroesophageal reflux disease who had negative upper gastroesophageal endoscopy and received the final diagnosis of functional dyspepsia (T0; n = 11). All the others resulted to be celiac with different degrees of villous atrophy (T3a, n = 3; T3b, n = 6; T3c, n = 7).

Genotyping of these 27 subjects resulted into 13 CC-homozygous, 12 CA-heterozygous and 2 AA-homozygous. The distribution of the DMT1 IVS4+44C>A genotypes in this cohort did not significantly differ from that found in the 387 celiacs (13-CC, 12-CA, 2-AA vs.161-CC, 193-CA, 33-AA; χ^2^ = 0.04, df = 2, *p* = 0.8) neither from that showed by the 164 healthy subjects (13-CC, 12-CA, 2-AA vs.66-CC, 81-CA, 17-AA; χ^2^ = 0.671, df = 2, *p* = 0.71). This cohort of 27 subjects for the mRNA study was in Hardy-Weinberg equilibrium (χ^2^ = 0.24, df = 2, *p* = 0.89). The stratification of villous atrophy degrees with respect to DMT1 IVS4+44C>A is reported in Table 4. The different degrees of atrophy did not show all of the possible 3 different IVS4+44C>A genotypes.

**Table 4:** Villous atrophy degree of 27 consecutive duodenal biopsies stratified according to DMT1 IVS+44C>A polymorphism.

Atrophy degree	DMT1 IVS+44C>A			*Row Total*
	CC	CA	AA	
**T0**	3 (11%)	11 (41%)	2 (7.50%)	11 (41%)
**T3a**	2 (7.50%)	3 (11%)	0 (0%)	3 (11%)
**T3b**	2 (7.50%)	6 (22%)	0 (0%)	6 (22%)
**T3c**	6 (22%)	7 (26%)	0 (0%)	7 (26%)
*Column Total*	13 (48%)	27 (100%)	2 (7.50%)	27 (100%)

#### Total DMT1 expression

**Fig 1:**
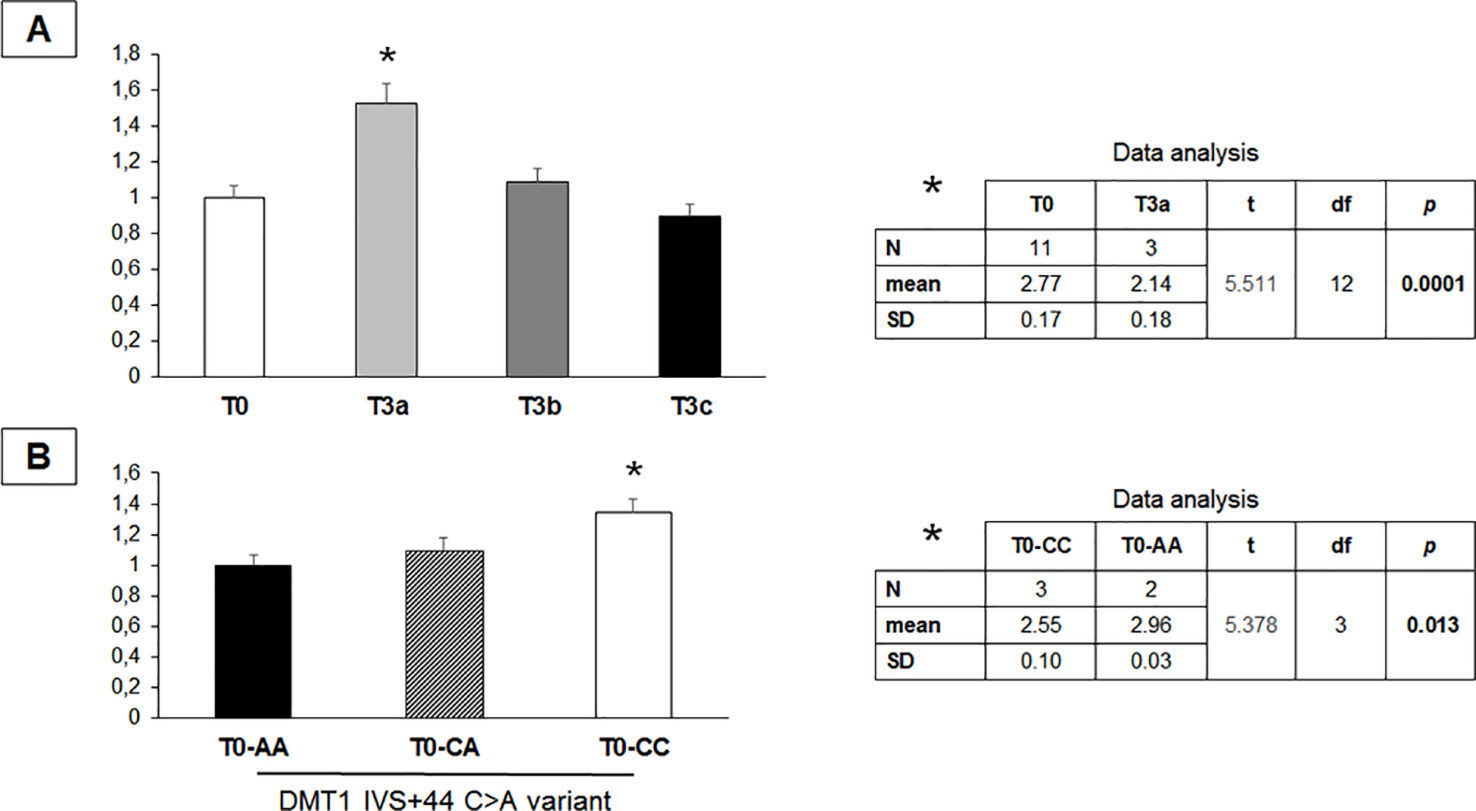
Total DMT1 expression in gastroesophageal biopsies from potential celiac children. **A)** Expression of total DMT1 mRNA from 27 gastroesophageal biopsies resulted into significant up-regulation of the iron transporter in the subgroup showing T3a degree of villous atrophy (n = 3) with respect to the subgroups with normal mucosa (T0 = 11). No significant difference in DMT1 expression was found in T3b (n = 6) and in T3c (n = 7) biopsies. On the right the result of the t-test between the ΔCt values for DMT1 expression (ΔCt = Ct _DMT1_- Ct _β-actin_) of T0 and T3a biopsies. **B)** Significant overexpression of total DMT1 transcript in null atrophy (T0) biopsies from CC-carriers with respect to those from AA-carriers, as shown by the t-test on the right between their ΔCt values for DMT1 expression. Data are represented as mean ± standard deviation of the fold change from at least three different assays performed in duplicate. The t-test has been used to determine the statistical significance between groups by using the ΔCt values of the DMT1 target and the β-actin reference, due to the small size number. A *p*-value less than 0.05 has been considered significant.

Total DMT1 expression resulted significantly increased in T3a biopsies with respect to those showing a normal mucosa (about 50%; *p* = 0.0001; Fig 1A). No significant change was observed in biopsies presenting more severe apical villous atrophy [T0 vs. T3b, t = 0.74 *p* = 0.47 (Table A in S2 File); T0 vs. T3c, t = 1.03, *p* = 0.32 (Table B in S2 File)].

Moreover, total DMT1 expression was higher in T0 biopsies from CC subjects compared to AA patients (about 40%; *p* = 0.013; Fig 1B) and to CA patients (S2 Table), whereas no significant difference of total DMT1 expression was found among the different genotypes in T3 biopsies (T3-CC vs. T3-CA, t = 1.32, *p* = 0.21; S3 Table).

### DMT1 ±IRE transcripts

Analysis of DMT1 +IRE and–IRE mRNA levels resulted in a +IRE/–IRE ratio less than one for 15 biopsies, expressing lower levels of +IRE transcript with respect to–IRE, and more than one for 12 biopsies, where the DMT1 +IRE transcript resulted to be the most expressed (S4 Table).

Stratifying the DMT1 +IRE/–IRE ratio for the DMT1 IVS4+44C>A variant, a significant association was found between the A-allele (due to the small sample size of CA and AA biopsies, they were combined together) and the DMT1 –IRE transcript (Table 5).

**Table 5:** DMT1 IVS+44C>A polymorphism is associated to DMT1 +IRE/–IRE transcript ratio.

DMT1	AA+CA	CC	*Row Total*	*df*	*p-value*
**+IRE/**–**IRE < 1**	11 (41%)	4 (15%)	15 (56%)	1	**0.021**
**+IRE/**–**IRE > 1**	3 (11%)	9 (33%)	12 (44%)		
*Column Total*	14 (52%)	13 (48%)	27 (100%)		
*Odds Ratio*	*95% LCL*	*95% UCL*	*chi-square*	*df*	*p-value*
8.2	1.45	46.86	6.238	1	**0.012**

Fisher’s exact test for determining the independence between the frequency of occurrence of a +IRE/–IRE transcript ratio less or more than one with respect to the DMT1 IVS+44C>A polymorphism.

Chi-square test for determining the *p*-value associated to the risk of expressing more DMT1 –IRE than DMT1 +IRE, conferred by the DMT1 IVS+44 A-allele.

Abbreviations: IRE, iron responsive element; + = present;– = absent; df, degrees of freedom; LCL, lower confidence limit; UCL, upper confidence limit. *p*<0.05 has been considered significant.

## Discussion

In this study, we investigated, for the first time, the role of the IVS4+44C>A variant and the +IRE/–IRE transcripts of the DMT1 iron transporter in increasing susceptibility to anemia in a cohort of Italian celiac children.

Patients with IDA at diagnosis were significantly younger than non-anemic patients, confirming that pediatricians, recognizing anemia as one of the first clinical manifestations of CD, are promptly induced to prescribe serological screening [9].

According to literature [29], and due to the low mean age of our patients, we found that the percentage of males was significantly higher in anemic respect to non-anemic patients. Indeed, as expected, the incidence of IDA in our cohort was higher in males when considering 4 years old children or younger (*p* = 0.0003), but not in children older than 4 years (*p* = 0.7) (Tables A and B in S3 File). Indeed, IDA was more frequent in females when considering children aged between 11–18 years (*p* = 0.006), probably due to the menstrual [29] (Table C in S3 File).

The majority of the body’s iron requirement is adsorbed from the lumen of the proximal small intestine. Therefore, it is reasonable that CD, damaging the critical area for iron intake, compromises iron absorption. It has also been shown that reduced expression of several proteins critical in iron uptake regulation may be responsible for anemia in CD [26, 30].

According to these observations, we explore the possible association between IDA in CD and a polymorphism of the DMT1 gene, encoding for the protein designated to uptake the iron into the enterocytes. We chose the intronic polymorphism IVS4+44C>A, since recent studies demonstrate that CC-carriers show high iron levels [20–24]. In accordance to the Turkish study [24], we found that iron levels, as well as Hb levels, MCV and SI, were significantly lower in AA patients. Indeed, the A-allele was more frequent in celiac patients with IDA than in not anemic celiac patients (42% *vs* 29%; *p* = 3.5x10^-4^), as well as the AA genotype (16% *vs* 5%; *p* = 2x10^-4^), with a significantly higher risk for AA-homozygous patients to develop IDA compared to CA and CC carriers (OR 3,7; CI 95%; 1.77–7.85; *p* = 2x10^-4^).

Interestingly, neither IDA neither DMT1 IVS4+44C>A variant were associated to the grade of apical surface atrophy.

A multivariate analysis confirmed that the risk for anemia in CD is related to DMT1 IVS4+44 AA genotype, not only more than that conferred by the mucosa damage, but also more than that given by the body mass index, considered as co-factor for the obesity-related effects on iron metabolism [31, 32].

When systemic iron requirements augment, the expression of DMT1 mRNA and protein in the duodenum is increased [33–35] to facilitate iron absorption. Accordingly, we found a significant increase of total DMT1 expression in biopsies with T3a villous atrophy, compared to not atrophic biopsies (about 50%), but not in worsened degrees of atrophy, such as T3b and T3c. This data confirms that a damage to apical villi induces a considerable up-regulation of DMT1 transcripts. In the meantime, it suggests that this up-regulation cannot fully counteract the further loss of functional absorbing tissue that parallels the worsening of the disease. Noticeably, total DMT1 was significantly higher also in T0 biopsies from CC-carriers compared to those from CA and AA subjects (about 40%), but not in atrophic biopsies from CC-carriers.

Therefore, at severe villous atrophy stage, the genotype cannot more exert significant effects in terms of total DMT1 expression, whereas is the atrophy for its own that seems to trigger the up-regulation of the total amount of DMT1 transcripts. Nevertheless, this mechanism could be a common feature of all the atrophy-related diseases and not only CD specific.

It has been shown that only the DMT1 +IRE transcript is up-regulated in the duodenum of iron deficient animals, suggesting that IREs mediate iron modulation of DMT1 mRNA stability, as it happens for transferrin receptor transcription, where IREs protect the mRNA against nucleolytic degradation [36–38]. Noteworthy, we observe that DMT1 IVS4+44 A-allele affects the ratio of the DMT1 +IRE/–IRE transcripts toward the–IRE, that lacking the iron-dependent motif, could not efficiently respond to iron status variations. Therefore, this finding suggests that DMT1 IVS4+44 A variant may dysregulate the iron-induced changes in DMT1 expression and, in turn, may limit the overexpression of the transporter that normally occurs to respond to iron starvation. Possibly, this mechanism is not able to impair iron absorption in physiological condition. Nevertheless, it could be exacerbated in the presence of severe villus atrophy despite of DMT1 overexpression. Therefore, it results ineffective in counteracting iron deficiency at severe stage of disease. Indeed, the villous atrophy seems to not exert any significant influence on DMT1 transcript variants, whereas the polymorphism significantly affects the +IRE/–IRE ratio. Thus, IDA associated to CD further confirms the importance of interaction between genetic and environmental components for the onset of specific clinical features and comorbidities related to the disease.

This study has some limitations. Firstly, our results cannot be generalized to the overall Italian population or to other ethnicity, since our cohort was only from Southern Italy. Moreover, the biopsies were not representative of all the possible combinations between the three DMT1 IVS4+44C>A genotypes and the different stages of villous atrophy. Larger prospective and functional studies are required to confirm our data. Finally, it is necessary to clarify the role of the DMT1 IVS4+44C>A polymorphism in iron absorption with regard to supplementation therapy in untreated as well as in treated CD patients, in order to personalize their management and follow-up.

In conclusion, taking into account these limitations, the data from this study suggest, for the first time, that CD may unmask the contribution of the DMT1 IVS4+44C>A polymorphism to the risk for IDA.

## Supporting information

S1 Table Clinical features of 387 Italian children with celiac disease. (PDF)

S2 Table Data analysis of total DMT1 expression in non-atrophic duodenal biopsies stratified according to DMT1 IVS+44C>A polymorphism. (PDF)

S3 Table Data analysis of total DMT1 expression in atrophic biopsies stratified according to DMT1 IVS+44C>A polymorphism. (PDF)

S4 Table Data analysis of ±IRE DMT1 expression in duodenal biopsies from 27 consecutive subjects. (PDF)

S1 File DMT1-IVS +44C>A polymorphism is associated to iron deficiency anemia in celiac disease. (PDF)

S2 File Data analysis of total DMT1 expression in atrophic vs. non-atrophic biopsies. (PDF)

S3 File Age-related association between iron deficiency anemia and gender in celiac Italian children. (PDF)

## References

[1] Rossi F, Bellini G, Tolone C, Luongo L, Mancusi S, Papparella A, et al The cannabinoid receptor type 2 Q63R variant increases the risk of celiac disease: implication for a novel molecular biomarker and future therapeutic intervention. Pharmacol Res. 2012;66(1):88–94. doi: 10.1016/j.phrs.2012.03.011 22465144 10.1016/j.phrs.2012.03.011

[2] Tolone C, Cirillo G, Papparella A, Tolone S, Santoro N, Grandone A, et al A common CTLA4 polymorphism confers susceptibility to autoimmune thyroid disease in celiac children. Dig Liver Dis. 2009;41(6):385–9. doi: 10.1016/j.dld.2008.09.001 18929517 10.1016/j.dld.2008.09.001

[3] Kivelä L, Kaukinen K, Huhtala H, Lähdeaho ML, Mäki M, Kurppa K. At-Risk Screened Children with Celiac Disease are Comparable in Disease Severity and Dietary Adherence to Those Found because of Clinical Suspicion: A Large Cohort Study. J Pediatr. 2017. 10.1016/j.jpeds.2016.12.077 28153477

[4] Halfdanarson TR, Litzow MR, Murray JA. Hematologic manifestations of celiac disease. Blood. 2007;109: 412–21. doi: 10.1182/blood-2006-07-031104 16973955 10.1182/blood-2006-07-031104 PMC1785098

[5] Annibale B, Severi C, Chistolini A, Antonelli G, Lahner E, Marcheggiano A, et al Efficacy of gluten-free diet alone on recovery from iron deficiency anemia in adult celiac patients. Am J Gastroenterol. 2001;96:132–7. doi: 10.1111/j.1572-0241.2001.03463.x 11197242 10.1111/j.1572-0241.2001.03463.x

[6] Hoffbrand AV. Anaemia in adult coeliac disease. Clin Gastroenterol. 1974;3:71–89. 4599261

[7] Unsworth DJ, Lock FJ, Harvey RF. Iron-deficiency anaemia in premenopausal women. Lancet. 1999;353:1100. 10.1016/s0140-6736(05)76459-x 10199377

[8] Bottaro G, Cataldo F, Rotolo N, Spina M, Corazza GR. The clinical pattern of subclinical/silent celiac disease: an analysis on 1026 consecutive cases. Am J Gastroenterol. 1999;94:691–6. doi: 10.1111/j.1572-0241.1999.00938.x 10086653 10.1111/j.1572-0241.1999.00938.x

[9] Smukalla S, Lebwohl B, Mears JG, Leslie LA, Green PH. How often do hematologists consider celiac disease in iron-deficiency anemia? Results of a national survey. Clin Adv Hematol Oncol. 2014;12:100–5. 24892255

[10] Gokce S, Arslantas E. Changing face and clinical features of celiac disease in children. Pediatr Int. 2015;57:107–12. doi: 10.1111/ped.12448 25040342 10.1111/ped.12448

[11] Ertekin V, Tozun MS, Küçük N. The prevalence of celiac disease in children with iron-deficiency anemia. Turk J Gastroenterol. 2013;24:334–8. 24254265 10.4318/tjg.2013.0529

[12] Cannizzaro R, Da Ponte A, Tabuso M, Mazzucato M, De Re V, Caggiari L, et al Improving detection of celiac disease patients: a prospective study in iron-deficient blood donors without anemia in north Italy. Eur J Gastroenterol Hepatol. 2014;26:721–4. doi: 10.1097/MEG.0000000000000100 24841904 10.1097/MEG.0000000000000100

[13] Andrews NC, Schmidt PJ. Iron homeostasis. Annu Rev Physiol. 2007;69:69–85. doi: 10.1146/annurev.physiol.69.031905.164337 17014365 10.1146/annurev.physiol.69.031905.164337

[14] Gunshin H, Mackenzie B, Berger UV, Gunshin Y, Romero MF, Boron WF, et al Cloning and characterization of a mammalian proton-coupled metal-ion transporter. Nature. 1997;388(6641):482–8. doi: 10.1038/41343 9242408 10.1038/41343

[15] Andrews NC. The iron transporter DMT1. Int J Biochem Cell Biol. 1999;31(10):991–4. 10582331 10.1016/s1357-2725(99)00065-5

[16] Fleming MD, Romano MA, Su MA, Garrick LM, Garrick MD, Andrews NC. Nramp2 is mutated in the anemic Belgrade (b) rat: evidence of a role for Nramp2 in endosomal iron transport. Proc Natl Acad Sci U S A. 1998;95(3):1148–53. 9448300 10.1073/pnas.95.3.1148 PMC18702

[17] Gruenheid S, Cellier M, Vidal S, Gros P. Identification and characterization of a second mouse Nramp gene. Genomics 1995;25(2):514–25. 7789986 10.1016/0888-7543(95)80053-o

[18] Canonne-Hergaux F, Gruenheid S, Ponka P, Gros P. Cellular and subcellular localization of the Nramp2 iron transporter in the intestinal brush border and regulation by dietary iron. Blood. 1999;93(12):4406–17. 10361139

[19] Hubert N, Hentze MW. Previously uncharacterized isoforms of divalent metal transporter (DMT)-1: implications for regulation and cellular function. Proc Natl Acad Sci U S A. 2002;99(19):12345–50. doi: 10.1073/pnas.192423399 12209011 10.1073/pnas.192423399 PMC129447

[20] Iolascon A, De Falco L. Mutations in the gene encoding DMT1: clinical presentation and treatment. Semin Hematol. 2009;46:358–70. doi: 10.1053/j.seminhematol.2009.06.005 19786204 10.1053/j.seminhematol.2009.06.005

[21] Przybyłkowski A, Gromadzka G, Członkowska A. Polymorphisms of metal transporter genes DMT1 and ATP7A in Wilson's disease. J Trace Elem Med Biol. 2014;28(1):8–12. doi: 10.1016/j.jtemb.2013.08.002 24120082 10.1016/j.jtemb.2013.08.002

[22] Wysokinski D, Danisz K, Blasiak J, Dorecka M, Romaniuk D, Szaflik J, et al An association of transferrin gene polymorphism and serum transferrin levels with age-related macular degeneration. Exp Eye Res. 2013;106:14–23. doi: 10.1016/j.exer.2012.10.003 23089144 10.1016/j.exer.2012.10.003

[23] He Q, Du T, Yu X, Xie A, Song N, Kang Q, et al DMT1 polymorphism and risk of Parkinson's disease. Neurosci Lett. 2011;501(3):128–31. doi: 10.1016/j.neulet.2011.07.001 21777657 10.1016/j.neulet.2011.07.001

[24] Kayaalti Z, Odabaşi M, Söylemezoğlu T. Genotype and allele frequencies of divalent metal transporter 1 polymorphism in Turkish population. Mol Biol Rep 2011;38(4):2679–84. doi: 10.1007/s11033-010-0410-x 21104143 10.1007/s11033-010-0410-x

[25] Howitt J, Putz U, Lackovic J, et al Divalent metal transporter 1 (DMT1) regulation by Ndfip1 prevents metal toxicity in human neurons. Proc Natl Acad Sci U S A. 2009;106(36): 15489–94. doi: 10.1073/pnas.0904880106 19706893 10.1073/pnas.0904880106 PMC2741278

[26] Barisani D, Parafioriti A, Bardella MT, Zoller H, Conte D, Armiraglio E, et al Adaptive changes of duodenal iron transport proteins in celiac disease. Physiol Genomics. 2004;17(3):316–25 doi: 10.1152/physiolgenomics.00211.2003 15054143 10.1152/physiolgenomics.00211.2003

[27] Husby S, Koletzko S, Korponay-Szabó IR, Mearin ML, Phillips A, Shamir R, et al; ESPGHAN Working Group on Coeliac Disease Diagnosis.; ESPGHAN Gastroenterology Committee.; European Society for Pediatric Gastroenterology, Hepatology, and Nutrition. European Society for Pediatric Gastroenterology, Hepatology, and Nutrition guidelines for the diagnosis of coeliac disease. J Pediatr Gastroenterol Nutr. 2012;54(1):136–60. doi: 10.1097/MPG.0b013e31821a23d0 22197856 10.1097/MPG.0b013e31821a23d0

[28] Beutler E, Waalen J. The definition of anemia: what is the lower limit of normal of the blood hemoglobin concentration? Blood. 2006;107(5):1747–50. doi: 10.1182/blood-2005-07-3046 16189263 10.1182/blood-2005-07-3046 PMC1895695

[29] Powers JM, Daniel CL, McCavit TL, Buchanan GR. Deficiencies in the Management of Iron Deficiency Anemia During Childhood. Pediatr Blood Cancer. 2016;63(4):743–5. doi: 10.1002/pbc.25861 26728130 10.1002/pbc.25861 PMC4755821

[30] Sharma N, Begum J, Eksteen B, Elagib A, Brookes M, Cooper BT, et al Differential ferritin expression is associated with iron deficiency in coeliac disease. Eur J Gastroenterol Hepatol. 2009;21:794–804. doi: 10.1097/MEG.0b013e328308676b 19404207 10.1097/MEG.0b013e328308676b

[31] del Giudice EM, Santoro N, Amato A, Brienza C, Calabrò P, Wiegerinck ET, et al Hepcidin in obese children as a potential mediator of the association between obesity and iron deficiency. J Clin Endocrinol Metab. 2009;94(12):5102–7. doi: 10.1210/jc.2009-1361 19850683 10.1210/jc.2009-1361

[32] Amato A, Santoro N, Calabrò P, Grandone A, Swinkels DW, Perrone L, et al Effect of body mass index reduction on serum hepcidin levels and iron status in obese children. Int J Obes. 2010;34(12):1772–4. 10.1038/ijo.2010.204 20877286

[33] Morgan EH, Oates PS. Mechanisms and regulation of intestinal iron absorption. Blood Cells Mol Dis. 2002;29(3):384–99. 12547229 10.1006/bcmd.2002.0578

[34] Gunshin H, Allerson CR, Polycarpou-Schwarz M, Rofts A, Rogers JT, Kishi F, et al Iron-dependent regulation of the divalent metal ion transporter. FEBS Lett. 2001;509:309–16. 11741608 10.1016/s0014-5793(01)03189-1

[35] Canonne-Hergaux F, Levy JE, Fleming MD, Montross LK, Andrews NC, Gros P. Expression of the DMT1 (NRAMP2/DCT1) iron transporter in mice with genetic iron overload disorders. Blood. 2001;29(3):384–99. 10.1182/blood.v97.4.1138 11159549

[36] Kong WN, Chang YZ, Wang SM, Zhai XL, Shang JX, Li LX, et al Effect of erythropoietin on hepcidin, DMT1 with IRE, and hephaestin gene expression in duodenum of rats. J Gastroenterol. 2008;43(2):136–43. doi: 10.1007/s00535-007-2138-5 18306987 10.1007/s00535-007-2138-5

[37] Wilkinson N, Pantopoulos K. The IRP/IRE system in vivo: insights from mouse models. Front Pharmacol. 2014;5:176 doi: 10.3389/fphar.2014.00176 25120486 10.3389/fphar.2014.00176 PMC4112806

[38] Casey JL, Di Jeso B, Rao K, Klausner RD, Harford JB. Two genetic loci participate in the regulation by iron of the gene for the human transferrin receptor. Proc Natl Acad Sci U S A. 1988;85(6):1787–91. 3162307 10.1073/pnas.85.6.1787 PMC279864

