# Supplementary material for: The DMT1 IVS4+44C>A polymorphism and the risk of iron deficiency anemia in children with celiac disease
Source: PLoS One. 2017 Oct 12;12(10):e0185822. doi: 10.1371/journal.pone.0185822 (PMC5638269; doi:10.1371/journal.pone.0185822)
Supplement: S1 Table — (PDF) [file pone.0185822.s001.pdf]

S1 Table

Clinical features of 387 Italian children with Celiac Disease

**Patients, n**	387
**Males, n (%)**<br>**Females, n (%)**	152 (39)<br>235 (61)
**Age, years, mean ± SD**<br>**(range)**	5.07 ± 4.00<br>(0.58-18.00)
**z-score BMI**	-0.005 ± 1.22
**Hb, g/dl, mean ± SD**	11.78 ± 1.44
**Hb < 3°, n (%)**<br>**Hb ≥ 3°, n (%)**	134 (35)<br>253 (65)
**MCV, fl, mean ± SD**	75.81 ± 7.47
**Sideremia, mcg/dl, mean ± SD**	57.44 ± 33.92
**Ferritin, ng/ml, mean ± SD**	21.91 ± 19.87
**Transferrin, mg/dl, mean ± SD**	315.45 ± 79.04
**Saturation Index, mean ± SD**	0.15 ± 0.09
**SI < 0.10, n (%)**<br>**SI ≥ 0.10, n (%)**	113 (37)<br>191 (63)
**Anti-tTg, U/ml, mean ± SD**	74.63 ± 89.68
**AGA IgA, U/ml, mean ± SD**	37.37 ± 63.26
**AGA IgG, U/ml, mean ± SD**	63.78 ± 116.34
**EMA, n (%)**<br>present<br>absent	 381 (98.5)<br>6 (1.5)
**Villous Atrophy, n (%)**<br>3a<br>3b<br>3c	 73 (19)<br>138 (36)<br>176 (45)

Abbreviations: SD, standard deviation; BMI, body mass index; Hb, haemoglobin; MCV, mean corpuscular volume; SI, saturation index; Anti-tTg,tissue transglutaminase antibodies; AGA, anti-gliadin antibody; Ig, immunoglobulin; EMA, anti-endomysial antibody.
