# Supplementary material for: The DMT1 IVS4+44C>A polymorphism and the risk of iron deficiency anemia in children with celiac disease
Source: PLoS One. 2017 Oct 12;12(10):e0185822. doi: 10.1371/journal.pone.0185822 (PMC5638269; doi:10.1371/journal.pone.0185822)
Supplement: S2 Table — (PDF) [file pone.0185822.s002.pdf]

S2 Table

Data analysis of total DMT1 expression in non-atrophic duodenal biopsies stratified according to DMT1 IVS+44C>A polymorphism

<table>
<tr><th colspan="6">Summary of Data</th></tr>
<tr><td></td><td>T0-CC</td><td>T0-CA</td><td>T0-AA</td><td colspan="2">Total</td></tr>
<tr><td>N</td><td>3</td><td>6</td><td>2</td><td colspan="2">11</td></tr>
<tr><td>∑ΔCt</td><td>7.640</td><td>16.940</td><td>5.920</td><td colspan="2">13.560</td></tr>
<tr><td>Mean</td><td>2.547</td><td>2.823</td><td>2.960</td><td colspan="2">2.712</td></tr>
<tr><td>$\sum \Delta Ct^2$</td><td>19.477</td><td>47.868</td><td>17.524</td><td colspan="2">37.001</td></tr>
<tr><td>SD</td><td>0.101</td><td>0.090</td><td>0.028</td><td colspan="2">0.238</td></tr>
<tr><th colspan="6">Result Details</th></tr>
<tr><td>Source</td><td>SS</td><td>df</td><td>MS</td><td>F</td><td>p</td></tr>
<tr><td>Between-treatments</td><td>0.239</td><td>2</td><td>0.119</td><td>15.457</td><td>0.012</td></tr>
<tr><td>Within-treatments</td><td>0.062</td><td>8</td><td>0.008</td><td></td><td></td></tr>
<tr><td>Total</td><td>0.301</td><td>10</td><td></td><td></td><td></td></tr>
</table>

The one-way ANOVA was used to analyze the difference of total DMT1 expression in non- atrophic duodenal biopsies with respect the DMT1 IVS+44C>A variant by using the real time-derived ΔCt values ($Ct_{DMT1} - Ct_{\beta\text{-actin}}$). $p<0.05$ has been considered significant.
Abbreviations: T0 = null villous atrophy degree; SD, standard deviations; SS, sum of squares, df, degrees of freedom; MS, mean square.
