# Supplementary material for: The DMT1 IVS4+44C>A polymorphism and the risk of iron deficiency anemia in children with celiac disease
Source: PLoS One. 2017 Oct 12;12(10):e0185822. doi: 10.1371/journal.pone.0185822 (PMC5638269; doi:10.1371/journal.pone.0185822)
Supplement: S3 Table — (PDF) [file pone.0185822.s003.pdf]

S3 Table

Data analysis of total DMT1 expression in atrophic biopsies stratified according to DMT1 IVS+44C>A polymorphism

	T3-CC	T3-CA	t	df	*p*
**N**	10	6	1.32	14	**0.21**
**mean**	3.07	2.25			
**SD**	1.30	0.98			

The t- test was used to analyze the difference of total DMT1 expression in atrophic duodenal biopsies from homozygous CC and heterozygous CA subjects by using the real-time derived ΔCt values (Ct_{DMT1} – $Ct_{\beta\text{-actin}}$). Biopsies from AA homozygous subjects were not represented in our sample. Abbreviations: T3, severe villous atrophy degree; df, degrees of freedom; SD, standard deviations.
