# Supplementary material for: The DMT1 IVS4+44C>A polymorphism and the risk of iron deficiency anemia in children with celiac disease
Source: PLoS One. 2017 Oct 12;12(10):e0185822. doi: 10.1371/journal.pone.0185822 (PMC5638269; doi:10.1371/journal.pone.0185822)
Supplement: S4 Table — (PDF) [file pone.0185822.s004.pdf]

S4 Table

Data of ±IRE DMT1 expression in duodenal biopsies from 27 consecutive subjects

mRNA	DMT1 IVS+44C>A	+IRE/-IRE	+IRE/-IRE (pixel)	+IRE/S18 (pixel)	-IRE/S18 (pixel)	atrophy (T-degree)
1313	CC	1	1.6577453	40.88	24.66	3c
1314	CC	1	1.0274928	250.4	243.7	3b
1315	CA	0	0.0688096	10.81	157.1	3b
1316	CA	0	0.2185829	51.52	235.7	0
1317	CC	1	1.0851342	165.7	152.7	3c
1318	CC	0	0.1960088	44.2	225.5	3c
1319	CA	1	1.5476683	29.87	19.3	0
1320	CC	1	4.9936908	31.66	6.34	3b
1321	CA	1	1.5534179	37.95	24.43	0
1322	CC	1	1.1502789	32.99	28.68	3c
1323	CA	0	0.9325153	30.4	32.6	3c
1324	CC	1	2.3025210	49.32	21.42	3a
1325	CA	0	0.0640932	8.8	137.3	3b
1326	CA	0	0.6831357	20.74	30.36	0
1327	CA	0	0.7111592	32.82	46.15	3a
1328	AA	0	0.8080905	67.12	83.06	0
1329	CC	1	1.1012978	104.37	94.77	3c
1330	CC	0	0.1872326	56.9	303.9	3c
1333	CC	0	0.3879186	105.06	270.83	0
1334	CA	0	0.6508142	201.03	308.89	3b
1335	CA	0	0.6825865	242.68	355.53	3b
1336	CC	1	1.6468023	33.99	20.64	3a
CTRL0	CC	1	1.2618173	278.42	220.65	0
CTRL1	AA	0	0.6520718	211.03	323.63	0
CTRL2	CA	0	0.6487497	258.41	398.32	0
CTRL3	CC	0	0.4727406	128.42	271.65	0
CTRL4	CA	1	1.7455115	283.89	162.64	0

Quantification of the DMT1 +IRE and –IRE bands with respect to the S18 bands, obtained by semi-quantitative PCR. After normalization with respect to S18, the ratio between +IRE/18S and –IRE/18S values was calculated and categorized as less or more than one. Pixels were quantized by using the quantization software Quantity One - 4.6.5 (Bio-Rad, Hercules, CA, USA).
