# Supplementary material for: The DMT1 IVS4+44C>A polymorphism and the risk of iron deficiency anemia in children with celiac disease
Source: PLoS One. 2017 Oct 12;12(10):e0185822. doi: 10.1371/journal.pone.0185822 (PMC5638269; doi:10.1371/journal.pone.0185822)
Supplement: S1 File — (PDF) [file pone.0185822.s005.pdf]

S1 File

DMT1 IVS+44C>A polymorphism is associated to iron deficiency anemia in celiac disease

A

Increased risk for presenting anemia in celiac DMT1 IVS+44 A-allele carriers

	**AA+CA**	**CC**	*chi-square*	*df*	*p-value*	*Odds Ratio*	*95% LCL*	*95% UCL*
**IDA**	91 (40%)	43 (27%)	4.812	1	**0.006**	**1.85**	1.19	2.87
**non-IDA**	135 (60%)	118 (63%)						

B

Increased risk for presenting anemia in celiac DMT1 IVS+44 homozygous AA carriers

	**AA**	**CA+CC**	*chi-square*	*df*	*p-value*	*Odds Ratio*	*95% LCL*	*95% UCL*
**IDA**	21 (64%)	113 (32%)	13.414	1	**0.0002**	**3.73**	1.77	7.85
**non-IDA**	12 (36%)	241 (68%)						

Abbreviations: df, degrees of freedom; LCL, lower confidence limit; UCL, upper confidence limit; IDA, iron deficiency anemia. p<0.05 has been considered significant.
