# Supplementary material for: The DMT1 IVS4+44C>A polymorphism and the risk of iron deficiency anemia in children with celiac disease
Source: PLoS One. 2017 Oct 12;12(10):e0185822. doi: 10.1371/journal.pone.0185822 (PMC5638269; doi:10.1371/journal.pone.0185822)
Supplement: S2 File — (PDF) [file pone.0185822.s006.pdf]

S2 File

Data analysis of total DMT1 expression in atrophic vs. non-atrophic biopsies

A

Data analysis of total DMT1 expression in T0 vs. T3b biopsies

	**T0**	**T3b**	**t**	**df**	***p***
**N**	11	6	0.742	15	**0.47**
**mean**	2.77	2.52			
**SD**	0.17	1.13			

B

Data analysis of total DMT1 expression in T0 vs. T3c biopsies

	**T0**	**T3c**	**t**	**df**	***p***
**N**	11	7	1.03	16	**0.32**
**mean**	2.77	3.23			
**SD**	0.17	1.47			

The t- test was used to analyze the difference of total DMT1 expression between non-atrophic (T0) duodenal biopsies with respect to T3b (A) or T3C (B) villous atrophy degree, by using the real-time derived ΔCt values ($Ct_{DMT1} - Ct$ β-actin).
Abbreviations: df, degrees of freedom; SD, standard deviations.
