# Supplementary material for: The DMT1 IVS4+44C>A polymorphism and the risk of iron deficiency anemia in children with celiac disease
Source: PLoS One. 2017 Oct 12;12(10):e0185822. doi: 10.1371/journal.pone.0185822 (PMC5638269; doi:10.1371/journal.pone.0185822)
Supplement: S3 File — (PDF) [file pone.0185822.s007.pdf]

S3 File

Age-related association between iron deficiency anemia and gender in celiac Italian children

A) Celiac children ≤ 4 years old

	**M**	**F**	*chi-square*	*df*	*p-value*	*Odds Ratio*	*95% LCL*	*95% UCL*
**IDA**	50 (57%)	39 (32.5%)	12.832	1	**0.0003**	**2.81**	1.58	4.97
**non-IDA**	37 (43%)	81 (68.5%)						

B) Celiac children 11-18 years old

	**M**	**F**	*chi-square*	*df*	*p-value*
**IDA**	17 (26%)	28 (24%)	0.072	1	**0.79**
**non-IDA**	48 (74%)	87 (76%)			

C) Celiac children > 4 years old

	**M**	**F**	*chi-square*	*df*	*p-value*	*Odds Ratio*	*95% LCL*	*95% UCL*
**IDA**	2 (9%)	9 (45%)	7.406	1	**0.006**	**8.59**	1.57	46.88
**non-IDA**	21 (91%)	11 (55%)						

Abbreviations: M, males; F, females; df, degrees of freedom; LCL, lower confidence limit; UCL, upper confidence limit; IDA, iron deficiency anemia. $p<0.05$ has been considered significant.
